# Simulations of Fractures of Heterogeneous Orthotropic Fiber-Reinforced Concrete with Pre-Existing Flaws Using an Improved Peridynamic Model

**DOI:** 10.3390/ma15113977

**Published:** 2022-06-02

**Authors:** Luming Zhou, Shu Zhu, Zhende Zhu, Xinghua Xie

**Affiliations:** 1 Key Laboratory of Ministry of Education for Geomechanics and Embankment Engineering, Hohai University, Nanjing 210098, China; sk_zlm@163.com (L.Z.); zzdnj@hhu.edu.cn (Z.Z.); 2 Jiangsu Research Center for Geotechnical Engineering Technology, Hohai University, Nanjing 210098, China; 3 State Key Laboratory of Hydrology-Water Resources and Hydraulic Engineering, Nanjing Hydraulic Research Institute, Nanjing 210029, China; njxiexinghua@163.com

**Keywords:** peridynamics, fiber-reinforced concrete, orthotropy, crack propagation and coalescence, failure mode

## Abstract

The propagation and coalescence of cracks in fiber-reinforced concretes (FRCs) is the direct cause of instability in many engineering structures. To predict the crack propagation path and failure mode of FRCs, an orthotropic-bond-based peridynamic (PD) model was established in this study. A kernel function reflecting long-range force was introduced, and the fiber bond was used to describe the macroanisotropy of the FRC. The crack propagation process of the FRC plate with flaws was simulated under uniaxial tensile loading. The results showed that under homogeneous conditions, the cracks formed along the centerline of the isotropic concrete propagate in a direction perpendicular to the load. Under anisotropic conditions, the cracks propagate strictly in the direction of the fiber bond. The failure degree of the FRC increases with the increase in heterogeneity. When the shape parameter is 10 and the fiber bond is 0°, the failure mode changes from tensile to shear failure. When the fiber bond is 45°, the FRC changes from a state where outer cracks penetrate the entire specimen to a state where cracks coalesce at the middle. It was found that the improved model can effectively simulate the crack propagation processes of orthotropic FRC materials.

## 1. Introduction

As a kind of heterogeneous brittle material, the essence of concrete failure is the process of its internal microcracks’ initiation, propagation, and penetration to form macrocracks, leading to its failure [1]. Compared with ordinary concrete, fiber-reinforced concrete (FRC) has significantly better mechanical properties, so it has been widely used in construction engineering [2]. Therefore, it is of great significance to study the initiation, propagation, and coalescence of concrete cracks to ensure the stability of engineering structures.

Deng et al. [3] studied the failure characteristics of hybrid steel–polypropylene FRC under uniaxial cyclic tension. Zhang et al. [4] conducted Brazilian disc splitting tests on basalt FRC samples with different fiber volume fractions, and studied the effects of fiber content on tensile strength. Del prete et al. [5] studied the creep behavior of polypropylene FRC. Although a large number of laboratory tests, such as the above, have been employed to study the crack propagation and coalescence processes of FRC [3,4,5,6,7,8,9], there remain some difficulties in studying FRC fractures. (1) To obtain comprehensive and ideal results, it is necessary to perform multiple groups of parallel experiments, which require considerable time and cost. (2) Typically, it is difficult to determine the degree of heterogeneity of concrete specimens. (3) A tensile brittle fracture occurs in a very short time, and it is difficult to clearly and completely observe the propagation process of internal cracks in laboratory tests. (4) Research on the fracture mechanisms of FRC with pre-existing flaws is insufficient.

The numerical simulation method can effectively solve the above problems, which can be divided into two types: one based on continuum mechanics, and one based on discontinuum mechanics. The finite element method (FEM) [10] is a commonly used method based on continuum mechanics for simulating crack propagation. However, the governing equation of the FEM is derived from continuum mechanics, and the stress field at the crack tip is mathematically singular. The extended finite element method (XFEM) can simulate the crack propagation by introducing a local strengthening function. However, because of the partial strengthening of the adjacent elements where the crack tip is located, the establishment of the element decomposition method cannot be ensured, hindering the application of this method in the case of multi-crack propagation with complex morphologies [11]. Meshless methods (MLMs), such as smoothed-particle hydrodynamics (SPH) [12,13] and the element-free Galerkin (EFG) method [14], eliminate the mesh dependency. Compared with the XFEM, MLMs improve the continuity of the interpolation [15]; however, the shape functions in conventional SPH and EFG do not meet the nature of the Kronecker delta function, making it difficult to apply the essential boundary conditions [16].

Common non-continuous numerical methods include discontinuous deformation analysis (DDA), the discrete element method (DEM), and so on. In DDA, the calculation format is unified for continuous and discontinuous problems [17]. However, the prediction of the crack propagation path still depends on the grid cells’ division [18]. In the DEM approach, the concrete is composed via a process of particle accumulation and cementation. Although the DEM does not involve the establishment or selection of a complex macroscopic constitutive model, it is necessary to calibrate the macro- and microparameters, which influence the calculation efficiency and the accuracy of the results [19,20].

Silling et al. [21,22] proposed the theory of peridynamics (PD) and a corresponding numerical method to simulate the evolution of the processes of crack initiation, propagation, and coalescence in solid structures under a unified mathematical framework. Unlike the classical continuum mechanics involving the use of the displacement component derivative, PD is a nonlocal continuum mechanics theory. PD overcomes the shortcomings of crack-tip singularity, the need for external fracture criteria, the inability to simulate crack initiation, and the strong grid dependence. The PD theory can be divided into bond-based PD (BB-PD), ordinary-state-based PD (OSB-PD), and non-ordinary-state-based PD (NOSB-PD). Among them, the BB-PD theory is the earliest and most widely used method. The classical BB-PD model was established only for homogeneous isotropic materials. Although Oterkus et al. [23] proposed a PD model of a fiber-reinforced composite, the existing analytical models consider that the micromodulus controlling the force between material points is a constant independent of the distance—that is, the effects of the long-range force are not considered. Therefore, in this work, a PD model considering the effects of the changes in the relative distance between material points on the force between them is established.

AI et al. [24], Jin et al. [25], and Chen et al. [26] simulated the crack growth process of concrete and concrete composites based on the classical BB-PD model. Huang et al. [27] introduced a rate-dependent plastic damage model to improve the NOSB-PD model and simulate the real stress field in concrete. Zhang et al. [28] and Zhang et al. [29] used the fully discrete PD method to simulate the tensile fracture behavior of fiber-reinforced cementitious composites. Yaghoobi et al. [30] proposed a semi-discrete fiber-reinforced modeling method to improve the computational efficiency of the PD method. Ma et al. [31,32,33] studied the crack propagation behavior of asphalt concrete mixes, and explored a numerical method to predict the concrete creep under high sustained stresses. The above studies simulated the fracture modes of concretes in the presence of cracks. However, concrete is often a heterogeneous and anisotropic material (especially FRC) containing flaws such as cracks and holes simultaneously. There are few studies on the influence of different fiber directions on the crack evolution and failure modes of concrete under the condition of accurately controlling the degree of heterogeneity. Therefore, it is necessary to strengthen research on the crack propagation characteristics of heterogeneous anisotropic FRC with flaws.

Based on this, we improved the classical BB-PD constitutive model by introducing functions reflecting long-range force and short-range repulsive force to reflect the influence of the bond length. The fiber bond was used to reflect the macroanisotropy of FRC, and the Weibull distribution function was introduced to reflect the heterogeneity of the concrete. The feasibility of this model was verified by two cases. Finally, the crack propagation and coalescence processes of a concrete plate with prefabricated flaws were simulated under uniaxial tension, and the effects of heterogeneity and fiber direction on the FRC fracture mode were studied. Thus, this paper reveals the crack propagation law of heterogeneous anisotropic FRC, and provides a theoretical reference for ensuring the stability of engineering structures.

## 2. Classical BB-PD Theory

**Figure 1:**
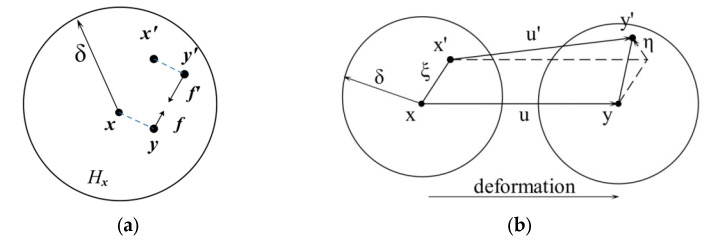
Illustration of the variables in the BB-PD model. (**a**) Horizon of x and pairwise force function; (**b**) Deformation diagram.

As shown in Figure 1a, the PD theory assumes that at any time *t*, any particle *x* in the object interacts with other particles *x’* in a certain area *H_x_* around it through the vector value function ***f***, which is called the pairwise force function, defined as follows:

(1) f=f(u(x′,t)−u(x,t),x′−x)

where ***u***(***x***,*t*) and ***u***(***x***′,*t*) are the displacement vectors of material points ***x*** and ***x***′, respectively.

According to Newton’s second law, the motion equation of the material point ***x*** at any time *t* is [21,22]:

(2) $$\rho \overset{\cdot \cdot }{u}(x,t)=\int_{H_{x}}f(u(x\prime ,t)-u(x,t),x\prime -x)dV_{x\prime }+b(x,t)$$

where *ρ* is the mass density, ***ü*** is the acceleration vector of ***x***, ***b*** is the applied physical density vector, *dV_x_*_′_ is the infinitesimal volume linked to point ***x***′, and *H_x_* is the horizon of ***x***. The concept of the horizon is as follows:

(3) $$H_{x}=H(x,\delta )=\left\{x\in R:\left\|x\prime -x\leq \delta \right\|\right\}$$

where *δ* is the radius of *H_x_*.

As shown in Figure 1b, ***ξ*** and ***η*** are the relative position vector and relative displacement vector of the points ***x*** and ***x***′, respectively.

(4) $$\left\{\begin{array}{l} \xi =x\prime -x\\ \eta =u(x',t)-u(x,t) \end{array}\right.$$

The BB-PD model uses stretch ***s*** to represent the bond deformation between material points, which is defined as follows [22]:

(5) $$s=\frac{\left\|\xi +\eta \right\|-\left\|\xi \right\|}{\left\|\xi \right\|}$$

A scalar function *μ* is introduced as the failure criterion of the bond:

(6) $$\mu (\xi ,x,t)=\left\{\begin{array}{ll} 1 & \;\mathrm{for}\;s\leq s_{0}\\ 0 & \;\mathrm{for}\;s>s_{0} \end{array}\right.$$

where *s*_0_ is the bond’s critical stretch; when the stretch of the bond exceeds *s*_0_, the bond will be broken irretrievably.

**Figure 2:**
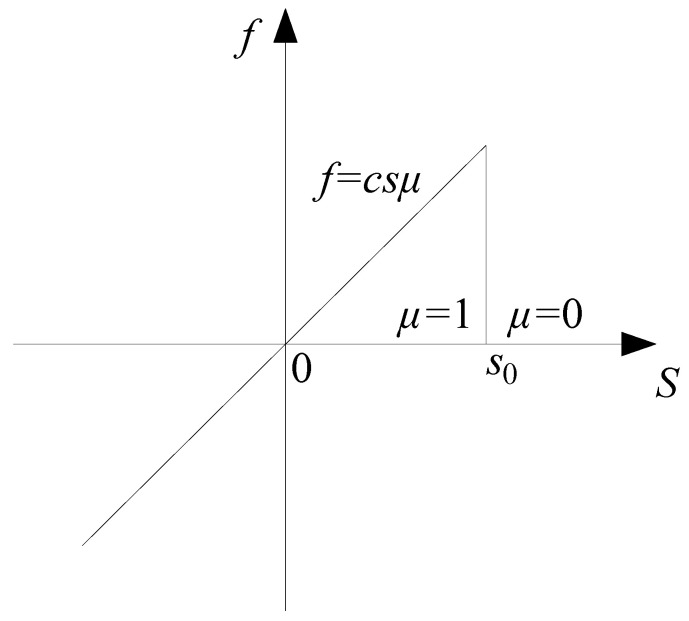
Classical BB-PD constitutive model.

The pairwise force function ***f*** is defined as follows [22]:

(7) $$f\left(\xi ,\eta \right)=\left\{\begin{array}{cc} \frac{\xi +\eta }{∥\xi +\eta ∥}c\left(\xi ,\delta \right)s\mu \left(\xi ,x,t\right), & ∥\xi ∥\leq \delta \\ 0, & ∥\xi ∥>\delta \end{array}\right.$$

where *c* is the micromodulus function. The classical BB-PD constitutive model is shown in Figure 2.

The pairwise force function ***f*** can be obtained from the derivative of micropotential energy *ω*:

(8) $$f(\xi ,\eta )=\frac{\partial \omega }{\partial \eta }(\xi ,\eta )$$

The strain energy density *W_PD_* at a material point ***x*** can be expressed as follows:

(9) $$W_{PD}=\frac{1}{2}\int_{H_{x}}\omega (\xi ,\eta )dV_{\xi }$$

where 1/2 means that the strain energy of the bond is evenly distributed at the material points on both ends of the bond. Under the same load, the strain energy density obtained from Equation (10) should be equal to that obtained from the classical continuum mechanics theory. Combined with Equations (8)–(10), the micromodulus *c* for a 2D plane stress problem can be expressed as follows [22]:

(10) $$c=\frac{9E}{\pi h\delta^{3}}$$

where *E* is the elastic modulus, and *h* is the material thickness. Notably, in the isotropic BB-PD model, the Poisson’s ratio of the plane stress problem is limited to 1/3.

The critical stretch *s_0_* is determined on the basis of the breaking energy *G*_0_, which can be expressed as follows:

(11) $$G_{0}^{}=2h\int_{0}^{\delta }\int_{z}^{\delta }\int_{0}^{\arccos z/\xi }w_{0}(\xi )\xi d\varphi d\xi dz=\frac{cs_{0}^{2}h\delta^{4}}{4}$$

(12) $$s_{0}^{}=\sqrt{\frac{4G_{0}^{}}{hc\delta^{4}}}$$

The PD theory has its own failure criteria; the damage is defined by considering the fracture of the bond at a point, and the local damage ***φ*** at material point ***x*** is defined as follows [22]:

(13) $$\varphi (x,t)=1-\frac{\int_{H_{x}}\mu (x\prime -x,t)dV_{x\prime }}{\int_{H_{x}}dV_{x\prime }}$$

## 3. Improved Orthotropic PD Model

In the classical PD model, the micromodulus coefficient *c* is considered a constant. On this basis, the kernel function correction term g(ξ,δ) proposed by Huang et al. [34] is introduced:

(14) $$g\left(\xi ,\delta \right)=\left\{\begin{array}{cc} {\left(1-{\left(\frac{\xi }{\delta }\right)}^{2}\right)}^{2} & \xi \leq \delta \\ 0 & \xi >\delta \end{array}\right.$$

This correction term reflects the weakening of the pairwise force when the distance between the material point ***x*** and any other material point in the horizon range of ***x***—that is, the size effect of the nonlocal long-range force—increases.

The pairwise force function is corrected to:

(15) $$f(\xi ,\eta )=\frac{\xi +\eta }{\left\|\xi +\eta \right\|}g(\xi ,\delta )c(\xi ,\delta )s\mu (\xi ,x,t)$$

**Figure 3:**
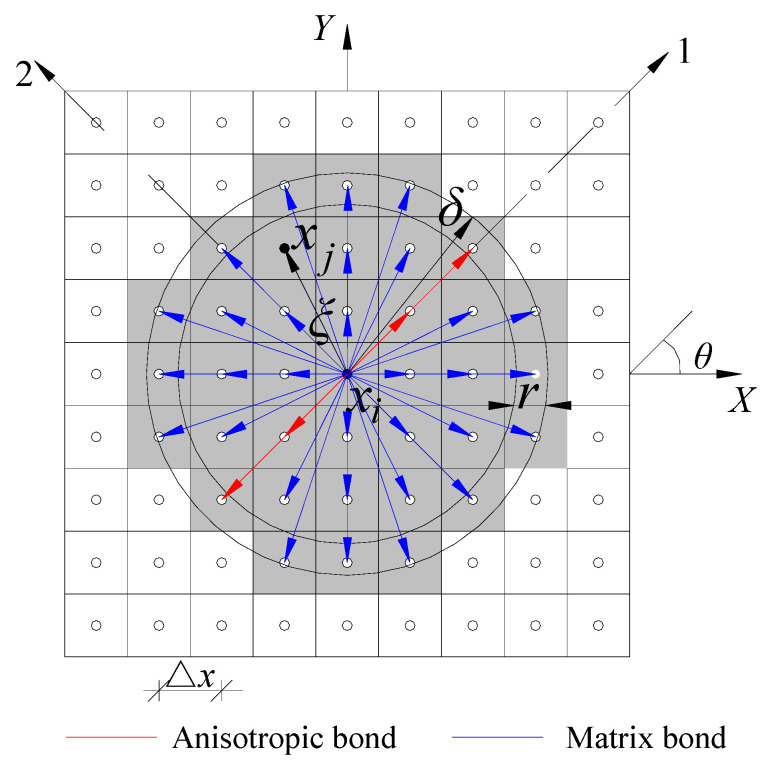
Schematic of the orthotropic PD model.

For 2D orthotropic FRC materials, it is assumed that the macroscopic mechanical properties (i.e., elastic modulus and Poisson’s ratio) and fracture criterion (i.e., critical stretch) in the angle *θ* direction (fiber direction) in the positive direction of the X-axis are different from those in other directions. As shown in Figure 3, the θ-oriented bonds are called fiber bonds, and the other arbitrarily oriented bonds are called matrix bonds. There are four independent material constants: elastic modulus *E*_1_ in the fiber bond direction, elastic modulus *E*_2_ in the matrix bond direction, Poisson’s ratio *ν*_12_, and shear modulus *G*_12_. The micromodulus *c* can be written as follows [23]:

(16) $$c=\left\{\begin{array}{cc} c_{m} & \varphi \neq \theta \\ c_{m}+c_{a} & \varphi =\theta \end{array}\right.$$

where *c_m_* and *c_a_* are the micromodulus of the matrix bond and fiber bond, respectively, and *φ* is the angle between any bond and the positive X-axis.

The strain energy density *W_PD_* in Equation (10) can be rewritten as follows [23]:

(17) $$W_{PD}=\frac{1}{2}\int_{H_{x}}\frac{c_{m}s_{m}{}^{2}\xi }{2}dV_{\xi }+\frac{1}{2}\sum_{q=1}^{Q}\frac{c_{a}s_{a}{}^{2}\xi_{a}}{2}V_{a}$$

where *Q* is the number of fiber bonds within the horizon range of material point *x*, *s_m_* and *s_a_* represent the stretch of the matrix bond and the fiber bond, respectively, *V_a_* is the volume occupied by another material point in the horizon range of the material point *x*, and the expression is as follows [23]:

(18) $$V_{a}=\frac{\pi h\delta^{2}}{N}$$

where *N* is the total number of material points in the horizon range of the point *x*.

For classical continuum mechanics, the stress–strain relationship of the orthotropic materials under the plane stress state can be expressed in the following matrix form:

(19) $$\left\{\begin{array}{c} \sigma_{11}\\ \sigma_{22}\\ \sigma_{12} \end{array}\right\}=\left[\begin{array}{ccc} D_{11} & D_{12} & 0\\ D_{12} & D_{22} & 0\\ 0 & 0 & D_{66} \end{array}\right]\left\{\begin{array}{c} \varepsilon_{11}\\ \varepsilon_{22}\\ \gamma_{12} \end{array}\right\}$$

where *D_ij_* is the stiffness matrix.

(20) $$\left\{\begin{array}{l} D_{11}=\frac{E_{1}}{1-\nu_{12}\nu_{21}},\;D_{12}=\frac{\nu_{12}E_{2}}{1-\nu_{12}\nu_{21}},\;D_{22}=\frac{E_{2}}{1-\nu_{12}\nu_{21}}\\ D_{66}=G_{12},\;1-\nu_{12}\nu_{21}>0,\;\frac{E_{1}}{\nu_{12}}=\frac{E_{2}}{\nu_{21}} \end{array}\right.$$

The strain energy density obtained from the classical continuum mechanics theory can be expressed as follows:

(21) $$W_{CM}=\frac{1}{2}(\sigma_{11}\varepsilon_{11}+\sigma_{22}\varepsilon_{22}+\sigma_{12}\gamma_{12})$$

The strain energy density obtained using Equations (17) and (21) is equal, as in the PD model of the orthotropic unidirectional plate deduced by Oterkus et al. [23]. Combined with Equation (14), the micromodulus of the modified orthotropic material PD model can be expressed as follows:

(22) $$\left\{\begin{array}{l} c_{m}=\frac{35E_{1}E_{2}{\left(1-{\left(\frac{\xi }{\delta }\right)}^{2}\right)}^{2}\;}{(E_{1}-\frac{1}{9}E_{2})\pi h\delta^{3}}\\ c_{a}=\frac{2E_{1}(E_{1}-E_{2})\;}{\left(E_{1}-\frac{1}{9}E_{2})(\sum_{q=1}^{Q}\xi_{a}V_{a}\right)} \end{array}\right.$$

**Figure 4:**
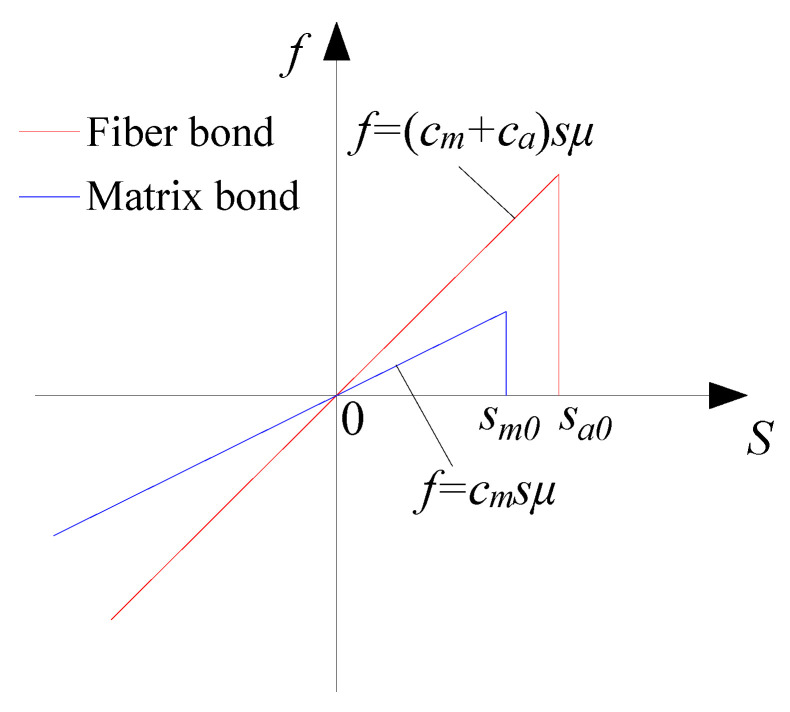
Orthotropic BB-PD constitutive model.

The orthotropic BB-PD constitutive model is shown in Figure 4. For orthotropic materials, the theoretical reference value of the critical elongation can be determined from a macro point of view [35,36]:

(23) $$\left\{\begin{array}{l} s_{m0}=T_{m}/E_{2}\\ s_{a0}=T_{a}/E_{1} \end{array}\right.$$

where *s_m_*_0_ and *s_a_*_0_ represent the critical stretch of the matrix bond and the fiber bond, respectively, while *T_m_* and *T_a_* represent the uniaxial tensile strength in the direction of the matrix bond and the fiber bond, respectively.

In addition, when the material points are in a state of compression, based on conventional continuum mechanics, two material points cannot overlap. Therefore, short-range repulsive forces should be introduced to prevent two or more material points from being at the same spatial position. According to Parks et al. [37], short-range repulsive forces ***f***_S_ can be expressed as follows:

(24) $$f_{S}(\xi ,\eta )=\frac{\xi +\eta }{\left\|\xi +\eta \right\|}\min\left\{0,\frac{cs}{\delta }\left\|\xi +\eta \right\|-\min[0.9\left\|x-x\prime \right\|,\;1.35(r+r\prime )]\right\}$$

Therefore, the motion equation of the improved BB-PD model can be rewritten as follows:

(25) $$\rho \overset{\cdot \cdot }{u}(x,t)=\int_{H_{x}}(f(u(x\prime ,t)-u(x,t),x\prime -x)+f_{S}(\xi ,\eta ))dV_{x\prime }+b(x,t)$$

## 4. Numerical Solution Method

The collocation method is used to solve Equation (17). As shown in Figure 3, the entire model is evenly discretized into multiple subdomains. The distance between any two center points is Δ*x.* At a certain time *t*, the material point *x_i_* interacts with the point *x_j_* within the horizon; thus, Equation (17) can be replaced using Riemann sums, as follows:

(26) $$\rho \overset{\cdot \cdot }{u_{i}^{t}}=\sum_{k}(f(\xi ,\eta^{t})+f_{S}(\xi ,\eta ))V_{k}+b_{i}^{t}$$

where *V_k_* is the volume of point *x_k_*. The material points on the horizon boundary are reduced proportionally on the basis of the relationship between the material points on the horizon boundary and the horizon radius. The point volume can be expressed as follows:

(27) $$V_{k}=\left\{\begin{array}{cc} {\left(\Delta x\right)}^{2}, & ∥\xi ∥\leq (\delta -r)\\ \left(\frac{\delta +r-∥\xi ∥}{2r}\right){\left(\Delta x\right)}^{2}, & (\delta -r)<∥\xi ∥\leq \delta \\ 0, & ∥\xi ∥>\delta \end{array}\right.$$

where *r* is half of the grid spacing Δ*x*.

Equation (26) can be solved using the explicit central difference scheme:

(28) $$\overset{\cdot \cdot }{u_{i}^{n}}=\frac{u_{i}^{n+1}-2u_{i}^{n}+u_{i}^{n-1}}{\Delta t^{2}}$$

where *n* is the number of time steps. According to Silling et al. [22], the value of time step Δ*t* should meet Equation (29):

(29) $$\left\{\begin{array}{l} \Delta t<\sqrt{\frac{2\rho }{\sum_{k}V_{k}\left|C(x_{k}-x_{i})\right|}}\\ C(\xi )=\frac{\partial f}{\partial \eta }(0,\xi ) \end{array}\right.$$

**Figure 5:**
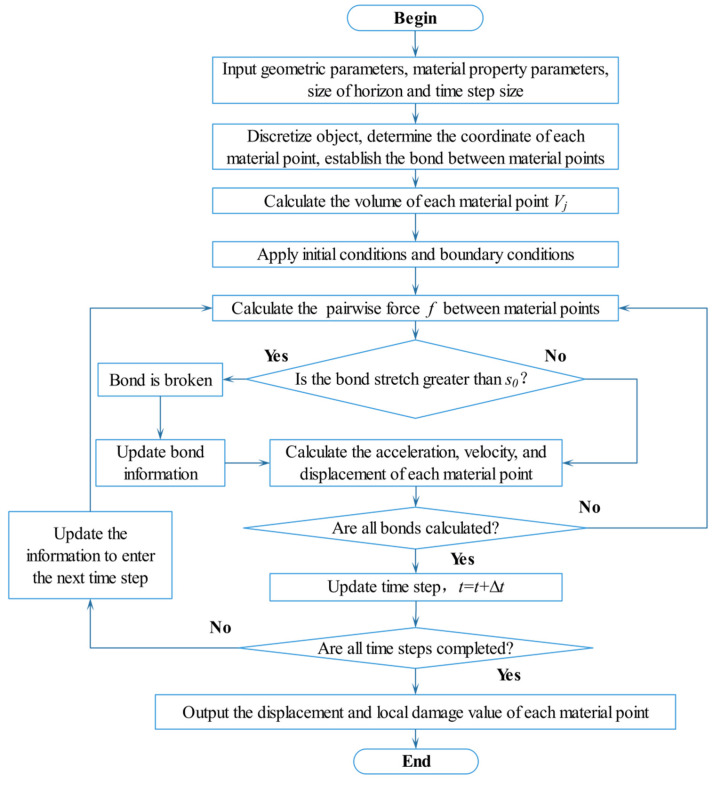
Simulation flowchart of the BB-PD model.

The Fortran language is used to carry out the above calculation process. Figure 5 shows the flowchart of the detailed work.

## 5. Model Validation

To validate the improved orthotropic BB-PD model in simulating FRC cracks, numerical examples of the elastic deformation of a cantilever beam and tensile failure of a fiber-reinforced orthotropic plate with pre-existing holes and fractures were simulated.

### 5.1. Elastic Deformation of a Cantilever Beam

**Figure 6:**
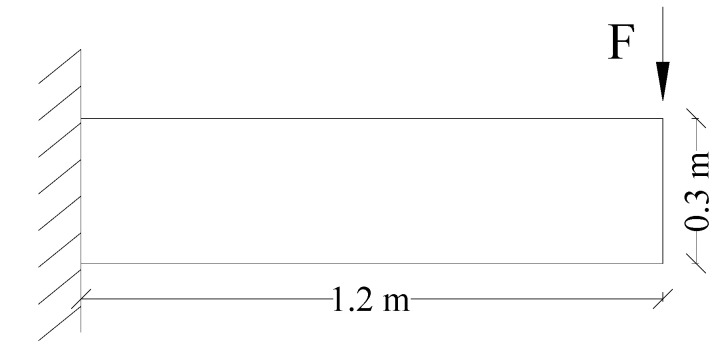
Geometric model of the elastic deformation of a cantilever beam.

To compare the performance of the improved PD model with that of the classical PD model, an example of the cantilever beam under concentrated load was applied, as shown in Figure 6. Compared with other models, such as joint beams, the analytical solution of this example is simpler, and the difference between the simulation results of the two PD models can be compared more intuitively. The cantilever beam size was 1.2 m × 0.3 m, the left boundary was fixed, and the right free end applied a downward concentrated load *F*. The elastic modulus was 19 Gpa, the Poisson’s ratio was 1/3, and the density was 2400 kg/m^3^. The PD numerical model was discretized to 90,000 material points, with a node spacing Δ*x* = 0.002 m, and the horizon *δ* = 3Δ*x*.

When considering the shear deformation, the analytical solution to the vertical displacement *u*_y_(*L*) of the midpoint on the right side of the elastic cantilever beam can be expressed as follows:

(30) $$u_{y}(L)=-F(\frac{L^{3}}{3EI}+\frac{L}{\kappa GA})$$

where *EI* and *GA* represent the bending stiffness and shear stiffness of the beam, respectively, and *κ* represents the shear correction factor, taken as 2/3.

**Figure 7:**
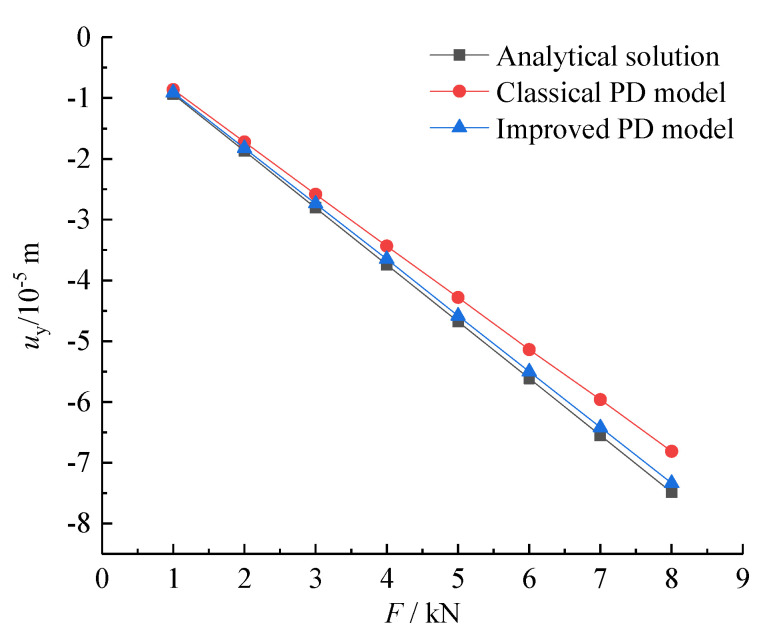
Load-displacement curves calculated by different methods.

During the simulation, a concentrated load increment of 1 kN was gradually applied as an external force to the material point at the rightmost boundary of the beam. After each load increment was applied, the load continued to increase after reaching the calculation stability for a period of time. Figure 7 shows the change in the vertical displacement at the midpoint of the free end of the beam obtained from the theoretical analytical solution, classical PD model, and improved PD model under different concentrated loads. Compared with the classical PD model, the improved model was closer to the analytical solution, and had a higher calculation accuracy.

### 5.2. Tensile Failure of a Fiber-Reinforced Orthotropic Plate with Pre-Existing Flaws

**Figure 8:**
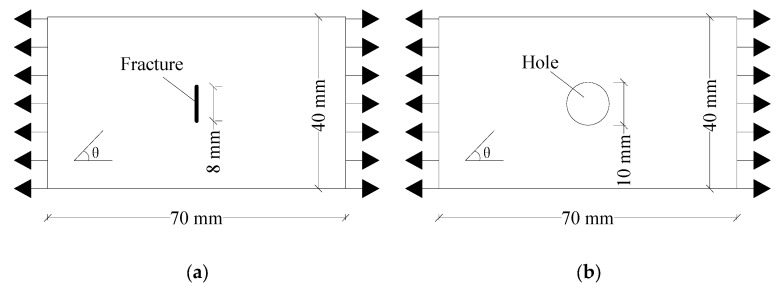
Geometric models of orthotropic thin plates with pre-existing flaws under tensile loads. (**a**) Flaw is a fracture; (**b**) Flaw is a hole.

As shown in Figure 8, the experimental model developed by Liu et al. [38] was used to verify the effectiveness of the improved PD model in predicting the crack propagation paths of fiber-reinforced orthotropic materials with flaws. The specimen was a unidirectional plate, which was prepared with M55J/Ag80 prepreg. The volume fraction of the fiber was 60% ± 3%, and the thickness of a single layer was 0.1 mm. The plate was 70 mm long and 40 mm wide. The prefabricated flaw was located at the center of the specimen, and the flaw included cracks and holes. The crack length was 8 mm, the inclination angle was 90°, and the hole diameter was 10 mm.

Hard fibers were embedded into the soft matrix materials at different angles to simulate the anisotropic properties. The fiber direction *θ* was the fiber bond direction. The elastic modulus in the fiber bond direction *E*_1_ = 106 Gpa, the elastic modulus in the matrix bond direction *E*_2_ = 8.5 Gpa, Poisson’s ratio *ν*_12_ = 1/3, and density *ρ* = 1801 kg/m^3^. The critical stretch *s_m_*_0_ = 0.02 and *s*_a0_ = 0.03. The specimen was discretized into 11,200 material points, with the node spacing Δ*x* = 0.5 mm, horizon *δ* = 3.015Δ*x*, and time step Δ*t* = 1 × 10^−8^ s. The tensile displacement load rate was 0.03 mm/s.

**Figure 9:**
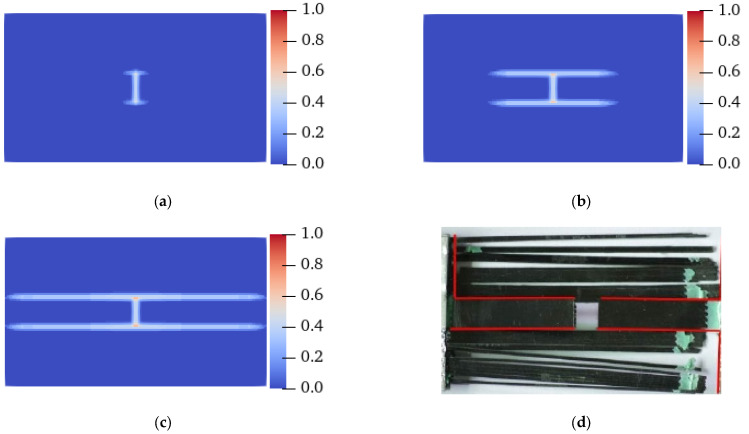
Experimental and PD simulation results of the tensile failure of a plate with fractures (*θ* = 0°). (**a**) 5.5 μs; (**b**) 8 μs; (**c**) 14 μs; (**d**) Experimental results [38].

**Figure 10:**
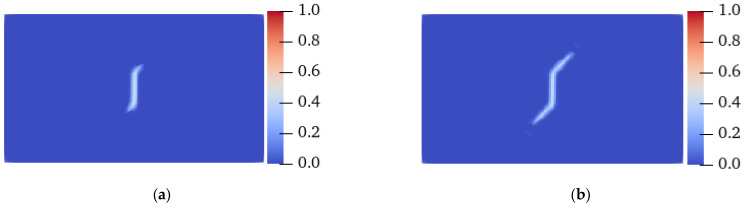

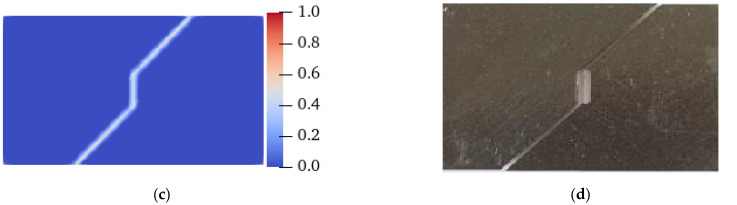
Experimental and PD simulation results of the tensile failure of a plate with fractures (*θ* = 45°). (**a**) 11 μs; (**b**) 12.5 μs; (**c**) 15.3 μs; (**d**) Experimental results [38].

Figure 9 and Figure 10 show the failure forms of thin plates with a fracture obtained from testing and PD simulation at *θ* = 0° and 45°, respectively. The PD simulation results show that when *θ* = 0° and 45°, the crack starts at 5.5 μs and 11 μs, respectively, and extends to the specimen boundary at 14 μs and 15.3 μs, respectively. Regardless of the angle *θ* of the fiber bond (0° or 45°), the crack initiation position is at the preset crack tip, and extends to both ends of the specimen along the *θ* direction. From the perspective of the PD theory, this phenomenon occurs because the critical stretch of the matrix bond is less than that of the fiber bond. With the expansion of damage, the broken matrix bond can no longer bear the load, so a part of the load is transferred to the fiber bond. As time progresses, the fiber bond gradually breaks, and the crack propagates along the fiber bond direction until the specimen fails.

**Figure 11:**
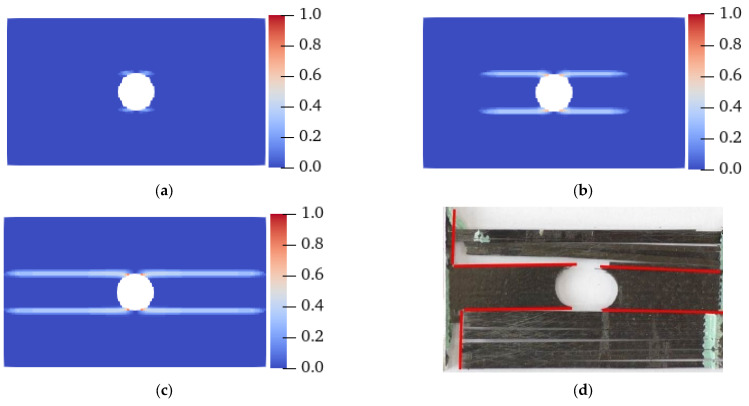
Experimental and PD simulation results of the tensile failure of a plate with holes (*θ* = 0°). (**a**) 5.5 μs; (**b**) 8 μs; (**c**) 15 μs; (**d**) Experimental results [38].

**Figure 12:**
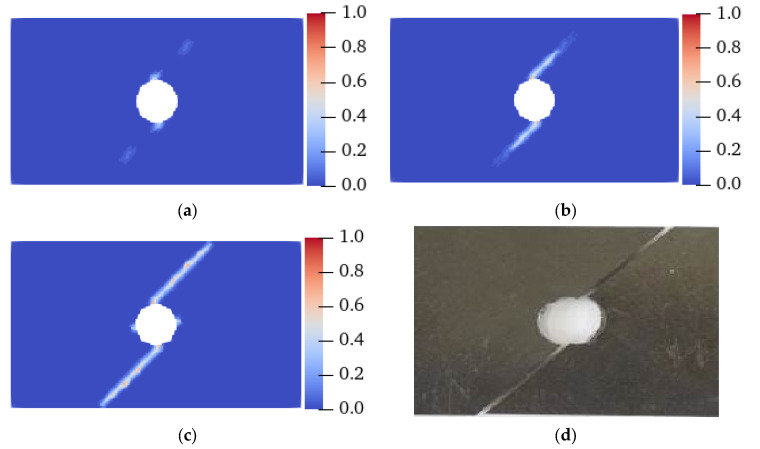
Experimental and PD simulation results of the tensile failure of a plate with holes (*θ* = 45°). (**a**) 12 μs; (**b**) 13.5 μs; (**c**) 15.3 μs; (**d**) Experimental results [38].

Figure 11 and Figure 12 show the failure modes of thin plates with holes obtained from the testing and PD simulation at *θ* = 0° and 45°, respectively. As shown, similar to the pre-existing fracture, under tensile loading, the crack starts at the upper and lower ends of the circular hole at 5.5 μs and 12 μs, respectively, and extends to both ends of the specimen along the direction of the fiber bond until the specimen is damaged at 15 μs and 15.3 μs, respectively. Regardless of whether the pre-existing flaw is a fracture or a hole, the crack propagates strictly in the *θ* direction of the fiber bond, and the simulated crack propagation path is highly consistent with the test, indicating that the improved PD model can effectively simulate the failure processes of the tensile fractures of anisotropic materials with pre-existing flaws. This also highlights the PD model’s advantages in simulating crack propagation and failure modes.

In this section, the ability of the improved PD model used to simulate the orthotropic fiber-reinforced materials is verified. In the reference experiment [38], the fiber directions were 0° and 45°. Therefore, fiber bonds in the directions of 0° and 45° were also selected for simulation.

## 6. Case Study

Since concrete is a typical heterogeneous material, the crack propagation conditions at each point in the concrete may be different. In most previous studies [39,40,41], normal distribution has been widely used to describe the heterogeneity of concrete. However, some studies have found that Weibull distribution can be used to describe the heterogeneity of concrete [42,43]. For example, Colman et al. [42] found that there was little difference between normal distribution and Weibull distribution in fitting the compressive strength of concrete. Tumidajski et al. [43] found that Weibull distribution can be applied to concrete compressive strength data based on chi-squared goodness-of-fit tests. Therefore, the Weibull distribution function can be used to realize the randomization of the mechanical properties of the materials to characterize the heterogeneity of concrete materials.

(31) $$W(p)=\frac{m}{p_{0}}{\left(\frac{p}{p_{0}}\right)}^{m-1}\exp[-{\left(\frac{p}{p_{0}}\right)}^{m}]$$

where *p* represents the distribution parameter value satisfied by each particle, *p*_0_ is the scale parameter representing the average value of the parameter *p*, and *m* is the shape parameter that determines the basic shape of the probability density function and reflects the homogeneity of the material structure.

It is assumed that the critical failure condition at each point of the concrete material obeys the Weibull distribution function with the mean value *s*_0_. Due to the inconsistent size of *s*_0_ of each particle, the mechanical properties of each point are affected. To ensure that the interaction force between two material points is equal, the critical stretch *s*_0_ was taken as the average value of the interacting material points, and its fracture judgment criterion can be expressed as *s* ≥ (*s*_0_(i) + *s*_0_(j))/2. The fractures of specimens with *m* values of 10, 20, and 30 were considered. Based on the characteristics of the Weibull distribution function, the lower the *m* value, the more discrete the distribution of the critical stretch of the concrete materials, and the greater the *m* value, the closer the critical stretch of the concrete materials to the mean value.

**Figure 13:**
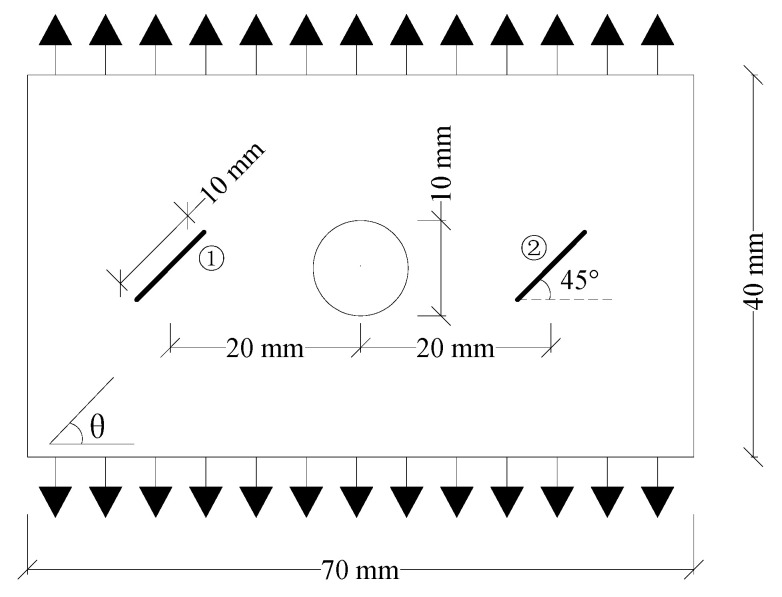
Geometric model of a concrete plate with flaws under tensile load.

As shown in Figure 13, the size of the rectangular concrete plate specimen was 70 mm × 40 mm. There was a circular hole at the center of the specimen, whose diameter was 10 mm. There were prefabricated fractures ① and ② with a length of 10 mm and an inclination of 45° on the left and right (LAR) sides of the hole’s center. The distance between the fracture center and the hole’s center was 20 mm. The specimen was dispersed into 70,000 material points, the spacing of nodes Δ*x* = 0.2 mm, the horizon *δ* = 3.015Δ*x*, and the time step Δ*t* = 1 × 10^−8^ s. The upper and lower (UAL) ends of the specimen were subjected to a tensile displacement load with a rate of 0.03 mm/s. Four cases were considered: homogeneous isotropy (without fiber), heterogeneous isotropy (without fiber), homogeneous anisotropy (with fiber), and heterogeneous anisotropy (with fiber).

### 6.1. Tensile Failure of an Isotropic Concrete Plate (without Fiber)

The elastic modulus of isotropic specimens *E* = 8.6 GPa, Poisson’s ratio *ν* = 1/3, and density *ρ* = 1800 kg/m^3^. The homogenization and critical stretch *s*_0_ were considered to obey the Weibull distribution, with a mean value of 0.02, and shape parameters (*m* values) of 10, 20, and 30.

**Figure 14:**
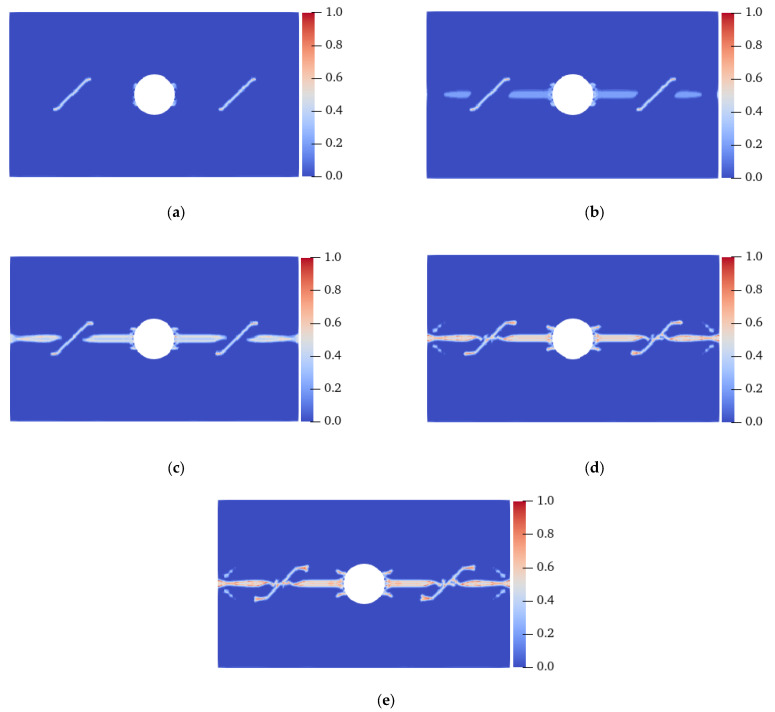
Crack propagation process of a homogeneous isotropic plate with flaws. (**a**) 8.8 μs; (**b**) 9.1 μs; (**c**) 9.5 μs; (**d**) 10.6 μs; (**e**) 11.3 μs.

Figure 14 shows the crack propagation process of the homogeneous isotropic plate with flaws under uniaxial tensile load. At 8.8 μs, the tensile crack initiated from the LAR ends of the prefabricated fractures ① and ② and the LAR sides of the hole. The cracks initiated on the UAL sides of the horizontal center line of the hole were symmetrically distributed, but the cracks were very small and slow to expand. At 9.1 μs, evident tensile cracks sprouted on the LAR sides of the horizontal centerline of the hole, and between the prefabricated fractures ① and ② and the LAR ends of the specimen. These cracks propagated in the direction perpendicular to the displacement load. At 9.5 μs, the tensile cracks on the LAR sides of the prefabricated fracture coalesced with the tensile cracks generated from the LAR ends of the specimen. At 10.6 s, the secondary cracks sprouted in the middle of the prefabricated fracture and coalesced with the tensile cracks on both sides of the hole’s centerline at 11.3 μs, and the specimen was destroyed. In the case of the homogeneous and isotropic plate, the concrete fracture was mainly due to the propagation and coalescence of tensile cracks on the LAR sides of the hole, the LAR sides of the specimen, and the center line of the middle area of the prefabricated fracture; however, the propagation rate of the earliest tensile cracks at both ends of the prefabricated fracture and on both sides of the hole was relatively slow.

**Figure 15:**
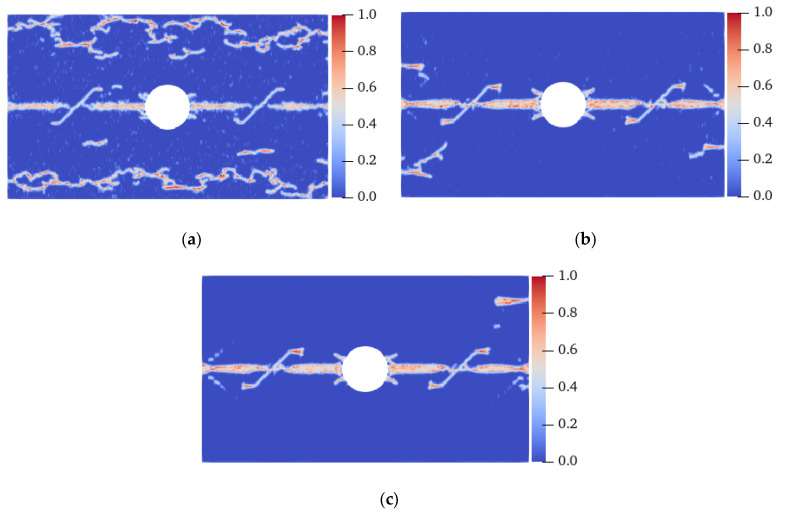
Failure modes of heterogeneous isotropic plates with flaws. (**a**) *m* = 10, 10.1 μs; (**b**) *m* = 20, 11.3 μs; (**c**) *m* = 30, 11.3 μs.

Figure 15 shows the final failure modes of isotropic specimens with different homogeneity. Ma et al. [36] believed that under uniaxial compression, the smaller the shape parameter *m*, the stronger the damage degree of the specimen. A similar conclusion was drawn in this study. When the shape parameter *m* was 10, unlike the fracture of the specimen from the horizontal centerline when it was isotropic, there were a large number of irregular tensile cracks with high local damage near the UAL ends of the specimen, and these cracks coalesced much earlier than the cracks on the centerline. When *m* was 20, there were evident irregular tensile cracks on the UAL sides of the left-end centerline and the lower side of the right-end centerline. When *m* was 30, except for the crack on the horizontal centerline, only the upper right end had an evident tensile crack. That is, under the same load conditions, the more heterogeneous the specimen, the more easily it was destroyed. An enhancement in the heterogeneity may lead to a change in the failure location; nevertheless, the failure mode is still tensile fracture.

### 6.2. Tensile Failure of an Anisotropic FRC Plate (θ = 0°)

In the case of anisotropy, the elastic modulus *E*_1_ in the direction of the fiber bond was 17.2 GPa, and the critical stretch *s_a_*_0_ = 0.03. The properties in the other directions were the same as those of the isotropic specimens. In addition to the homogeneous condition, it was considered that the critical stretch of the matrix bond *s_m_*_0_ and the critical stretch of the fiber bond *s_a_*_0_ obeyed the Weibull distribution, with mean values of 0.02 and 0.03, respectively, and shape parameter *m* values of 10, 20, and 30.

**Figure 16:**
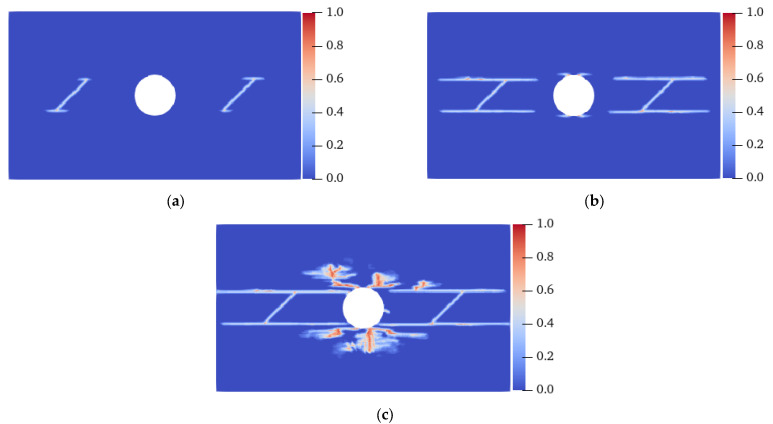
Crack propagation process of a homogeneous plate with flaws when the fiber bond was 0°. (**a**) 7.5 μs; (**b**) 13.5 μs; (**c**) 19.9 μs.

Figure 16 shows the crack propagation process of a plate with flaws when the fiber bond is 0° under homogeneous conditions. When the elastic modulus and critical stretch in the 0° direction were strengthened, the ends of the prefabricated fractures ① and ② started to crack first, and extended to the LAR sides at the same time in 7.5 μs, which was different from the isotropic situation, whose tensile cracks only sprouted in one direction. When the crack expanded for a period of time, the initiation of the crack occurred at the UAL sides of the hole at 13.5 μs. At 19.9 μs, the tensile crack at the lower part of the two prefabricated fractures extended inward to the hole and outward to both ends of the specimen, and the specimen was destroyed. Although tensile and shear cracks with serious local damage were produced at the UAL ends of the hole, it did not lead to a complete failure of the specimen.

**Figure 17:**
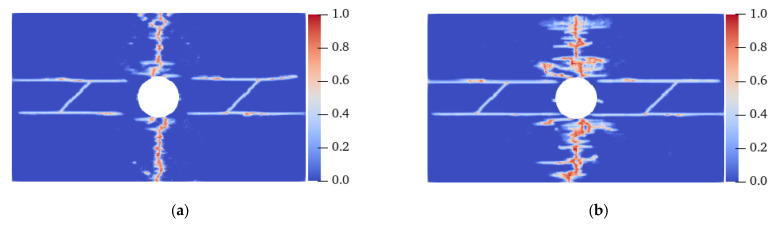

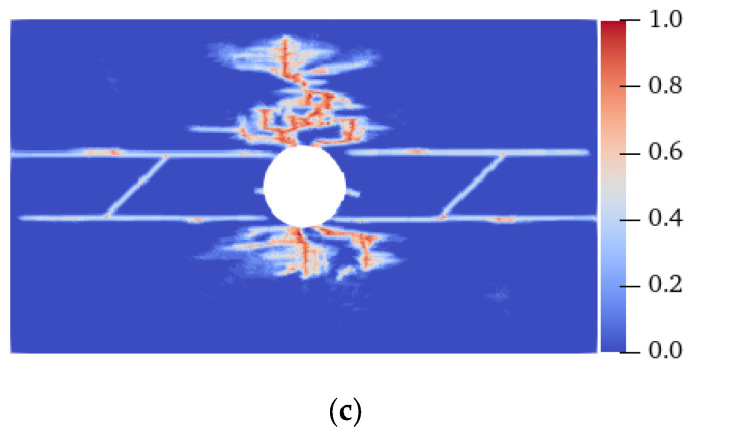
Failure modes of the heterogeneous plate with flaws when the fiber bond was 0°. (**a**) *m* = 10, 14.8 μs; (**b**) *m* = 20, 18.8 μs; (**c**) *m* = 30, 19.6 μs.

Figure 17 shows the final failure modes of specimens with different homogeneity at a 0° fiber bond. Under the condition of heterogeneity and anisotropy, the failure mode of the specimen changed: when *m* was 10, shear cracks with serious local damage were induced at the UAL ends of the vertical centerline of the hole, and the crack extended to the end of the specimen in 14.8 μs, resulting in the final failure of the specimen. When *m* was 20, the vertical propagation speed of the shear crack was relatively slow. At 18.8 μs, the shear and tensile cracks propagated almost to the end of the specimen simultaneously. When *m* was 30, the shear crack’s propagation speed was lower, and the failure mode was still a tensile failure. When the fiber bond was 0°, the strength in this direction was enhanced, resulting in the shear cracks generated at the UAL ends of the hole extending along the direction approximately parallel to the tensile load. When *m* was 10, the heterogeneity of the specimen was stronger than that when *m* was 20 and 30, resulting in faster shear crack propagation. Therefore, the failure mode of the specimen changed from tensile failure under isotropic conditions to shear failure. That is, for the anisotropic case, an enhancement in the specimen’s heterogeneity would aggravate the failure degree of the specimen and change the fracture position and failure mode.

### 6.3. Tensile Failure of an Anisotropic FRC Plate (θ = 45°)

**Figure 18:**
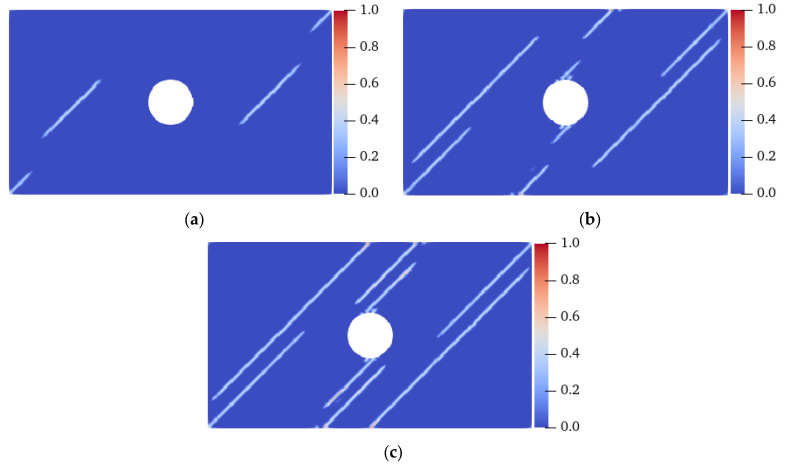
Crack propagation process of the homogeneous plate with flaws when the fiber bond was 45°. (**a**) 7.5 μs; (**b**) 11.0 μs; (**c**) 12.8 μs.

Figure 18 shows the crack propagation process of the plate with flaws when the fiber bond is 45° under homogeneous conditions. When the strength of the material in the 45° direction was strengthened, cracks propagating along the fiber bond direction appeared at the ends of the prefabricated fractures ① and ②, the bottom-left corner, and the top-right corner of the specimen at 7.5 μs. At 11 μs, cracks extending along the 45° direction also formed at the lower and upper ends of the vertical center line of the hole and the UAL ends of the specimen. At 12.8 μs, the cracks closest to the LAR ends of the specimen propagated to the end of the specimen, resulting in its failure. All of the cracks were symmetrically distributed throughout the entire process, and propagated in strict accordance with the direction of the fiber bond.

**Figure 19:**
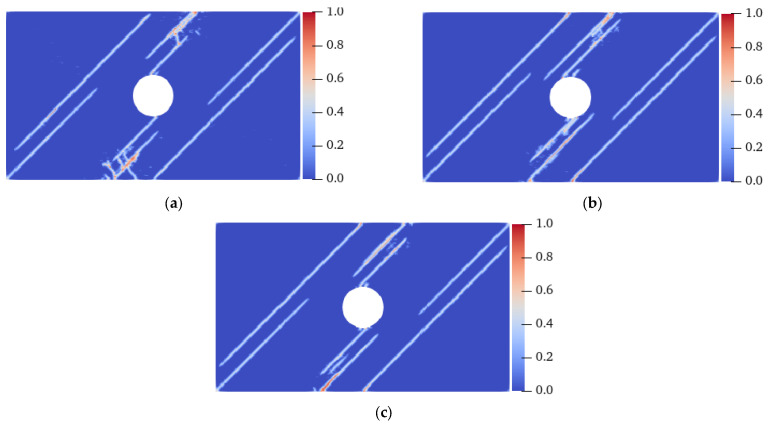
Failure modes of a heterogeneous plate with flaws when the fiber bond was 45°. (**a**) *m* = 10, 12.2 μs; (**b**) *m* = 20, 12.8 μs; (**c**) *m* = 30, 12.8 μs.

**Figure 20:**
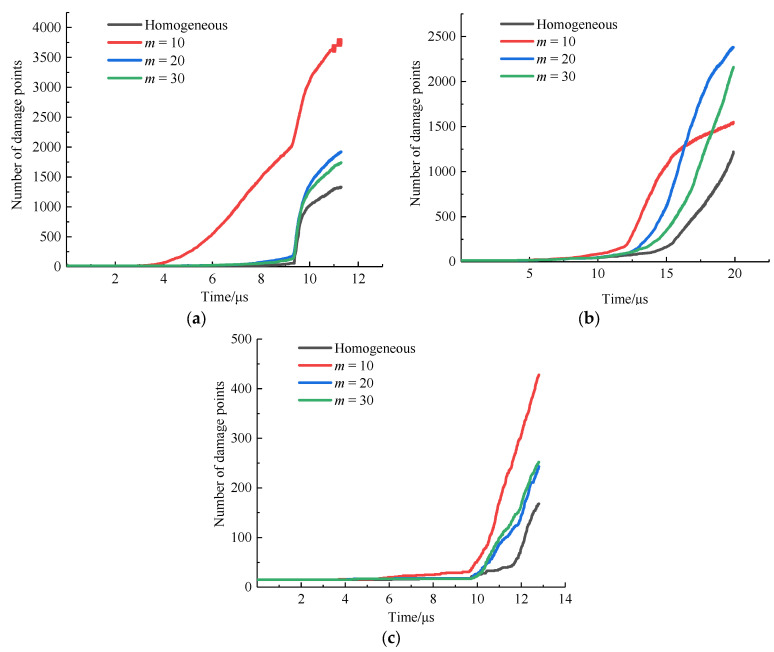
Curve of the change in damage degree. (**a**) Isotropy; (**b**) 0° fiber bond; (**c**) 45° fiber bond.

Figure 19 shows the final failure modes of specimens with different homogeneity at a 45° fiber bond. When *m* was 10, cracks with the same position and direction as those under the homogeneous condition were generated on the specimen; however, local damage was generated at the middle of the specimen and in the area between the cracks on the UAL sides of the hole. Local damage propagation caused the original inclined cracks to coalesce, resulting in a complete fracture before the inclined cracks near the LAR ends of the specimen extended to the end of the specimens. When the *m* values were 20 and 30, although local damage occurred at similar positions, the failure mode of the specimen was still the same as that of the homogeneous specimen. In combination with Figure 18 and Figure 20, it can be found that the degree of heterogeneity of the anisotropic specimen significantly affects the crack propagation path and the failure mode of the specimen relative to the isotropic specimen.

According to the above simulation results, it can be seen that the lower the *m* value, the more uneven the microscopic characteristics of the specimen, the worse the integrity and the quality of the concrete, and the easier it is to fracture. As a typical brittle material, the tensile strength of concrete is far less than its compressive strength. Under the tensile load, concrete fractures after only a small deformation, which is very similar to the strength characteristics of rock. Zhang et al. [44] established a PD model for rock-like materials based on Weibull distribution. It was found that the smaller the shape parameter, the more local damage occurs, and the easier the crack expands. Ma et al. [36] believed that the stronger the heterogeneity, the stronger the failure degree of the specimen, but the failure mode remains unchanged. The above studies are consistent with the simulation results in this work, which can verify the conclusions of this study to some extent.

### 6.4. Damage Degree Analysis

To more accurately compare the effects of heterogeneity and anisotropy on the damage degree of specimens, the variation over time in the number of material points with a local damage value above 0.5 in the process from the initial loading to the final failure of each case, under the same displacement loading condition, was determined. These points were called “damage points”, as shown in Figure 20.

Under isotropic conditions (Figure 20a), the numbers of damage points were 1330, 3800, 1920, and 1740 at 11.3 μs when the specimens were homogeneous, and the *m* values were 10, 20, and 30, respectively. The lower the m value, the easier the specimen was to fracture. The stronger the rock heterogeneity, the greater the increase in the damage degree. At the same time, when *m* was 10, the damage degree of the specimen was significantly higher than that of the other three cases. When the *m* values were 20 and 30, there was a small difference in the number of damage points, indicating that within a certain range, the fracture characteristics of the rock are less affected by the homogeneity. When the degree of heterogeneity exceeded a certain value, the damage degree of the rock significantly increased. When the fiber bond was 0° (Figure 20b) and m was 10, the specimen first completely fractured at 14.8 μs, and then the increase in the number of damage points decreased. This is because when the specimen’s strength in the 0° direction increases, the fracture mode of the specimen changes from tensile fracture to mixed tensile–shear fracture. As for the fiber bond of 45° (Figure 20c), the variation in the number of damage points was similar to that in the case of the isotropic bond.

Taking the homogeneous case as an example, the numbers of damage points in the specimens when the isotropic and fiber bonds were 0° and 45°, respectively, were 1330, 84, and 168, and the damage degree from high to low was as follows: isotropy > 45° fiber bond > 0° fiber bond. On the one hand, the strength in the fiber bond direction increased; therefore, it was more difficult to damage the material. On the other hand, when the fiber bonds were 0° and 45°, the angles between the fiber bond and the tensile load were 90° and 45°, respectively. When the angle was 90°, the component force in the direction of the fiber bond was lower; therefore, the damage degree of the specimen was lower.

## 7. Conclusions

In this study, an improved orthotropic PD model was established, and the corresponding micromodulus was derived. On this basis, the crack propagation process and failure mode of the heterogeneous anisotropic FRC specimens with flaws under tension were studied. The main conclusions are as follows:

(1) Comparing the analytical solution to the cantilever beam deformation and the experimental results of a fiber-reinforced orthotropic plate with flaws, it was verified that the improved PD model could effectively simulate the failure process of anisotropic FRC materials with flaws.
(2) Under isotropic conditions, the stronger the specimen’s heterogeneity, the more severe the failure degree, but the failure mode remained unchanged as tensile failure.
(3) Under orthotropic conditions, the specimen changed from tensile failure to shear failure when the fiber bond was 0°. When the fiber bond was 45°, the cracks propagated in strict accordance with the direction of the fiber bond, eventually forming sections that ran through the entire specimen, and leading to its failure.
(4) The stronger the concrete’s heterogeneity, the greater the increase in the damage degree. In the range of 0°–90°, the greater the angle between the fiber bond and the tensile load, the lower the damage degree of the specimen.
